# Supplementary material for: Pseudo-spin–valley coupled edge states in a photonic topological insulator
Source: Nat Commun. 2018 Aug 2;9:3029. doi: 10.1038/s41467-018-05408-w (PMC6072787; doi:10.1038/s41467-018-05408-w)
Supplement: Supplementary file 1 — Supplementary Information [file 41467_2018_5408_MOESM1_ESM.pdf]

Supplementary Information for

“Pseudospin-valley coupled edge states in a photonic topological insulator”

Kang et al.

Supplementary Note 1 – Derivation of the low-energy effective Hamiltonian

Pseudo spin topological insulator created by bianisotropic response

A triangular lattice of conducting rods with a broken symmetry in the cylindrical direction (z direction) is made by pushing the concentric collars away from the center of the copper plates of the metawaveguide, as described in the main text. As a result, the electric and magnetic dipoles are coupled in such way that cross terms between the electric and magnetic field arise in Maxwell's equations as follows:

$$\nabla \times \mathbf{E} = -ik_0(\hat{\mu}\mathbf{H} + \hat{\chi}\mathbf{E}) \tag{1}$$

$$\nabla \times \mathbf{H} = ik_0(\hat{\varepsilon}\mathbf{E} + \hat{\chi}^{\dagger}\mathbf{H}), \tag{2}$$

where $k_0 = \omega/c$ is the wavenumber in free space, ω is the angular frequency, and c is the speed of light. The constitutive tensors of the material are defined as follows:

$$\hat{\varepsilon} = \begin{pmatrix} \varepsilon_{\perp} & 0 & 0 \\ 0 & \varepsilon_{\perp} & 0 \\ 0 & 0 & \varepsilon_{zz} \end{pmatrix}, \quad \hat{\mu} = \begin{pmatrix} \mu_{\perp} & 0 & 0 \\ 0 & \mu_{\perp} & 0 \\ 0 & 0 & \mu_{zz} \end{pmatrix}, \quad \hat{\chi} = \begin{pmatrix} 0 & i\chi & 0 \\ -i\chi & 0 & 0 \\ 0 & 0 & 0 \end{pmatrix}, \tag{3}$$

where we assume $\hat{\varepsilon} = \hat{\mu}$ and χ are purely real. Ignoring propagation in the z direction, a reduced set of equations can be obtained for TM-like and TE-like modes coupled to each other by bianisotropy

$$\left(k_0^2\varepsilon_{zz} + \partial_x \mathrm{e} \partial_x + \partial_y \mathrm{e} \partial_y\right) E_z = -i\left(\partial_x \Delta \partial_y + \partial_y \Delta \partial_x\right) H_z, \tag{4}$$

$$\left(k_0^2\mu_{zz} + \partial_x m \partial_x + \partial_y m \partial_y\right) H_z = -i\left(\partial_x \Delta \partial_y + \partial_y \Delta \partial_x\right) E_z, \tag{5}$$

where we use the following notation $m = \dfrac{\mu_{\perp}}{\mu_{\perp}\varepsilon_{\perp} - \chi^2}$, $\mathrm{e} = \dfrac{\varepsilon_{\perp}}{\mu_{\perp}\varepsilon_{\perp} - \chi^2}$, and $\Delta = \dfrac{\chi}{\mu_{\perp}\varepsilon_{\perp} - \chi^2}$.Taking into account the equality $\mathrm{e} = m$, pseudo spin vectors are defined as $\psi^{\uparrow,\downarrow} = E_z \pm H_z$. Consequently, two independent equations for pseudo-spin-up and pseudo-spin-down states are obtained

$$\left(k_0^2\varepsilon_{zz} + \partial_x m \partial_x + \partial_y m \partial_y\right) \psi^{\uparrow,\downarrow} = \mp i\left(\partial_x \Delta \partial_y + \partial_y \Delta \partial_x\right) \psi^{\uparrow,\downarrow}, \tag{6}$$

Specifically, we consider here the case of the pseudo-spin-up state. The case of the pseudo-spin-down state is obtained in similar fashion. We assume ε_{zz} does not vary in space and is equal to ε in Supplementary Eq. (6). Using Bloch's theorem in this lattice, the field $\psi^{\uparrow}$ and the constitutive parameters are expanded in a Fourier series as

$$\psi^{\uparrow} = \sum_G \psi^{\uparrow}_{\mathbf{G}} e^{i(\mathbf{G}+\mathbf{q})\cdot\mathbf{r}}, \tag{7}$$

$$\{m, \Delta\} = \sum_G \{m_{\mathbf{G}}, \Delta_{\mathbf{G}}\} e^{i\mathbf{G}\cdot\mathbf{r}}, \tag{8}$$

The Fourier coefficients are given by

$$\{m, \Delta\}_{ij} = \frac{1}{S_0} \int\limits_{\text{u.c.}} \{m, \Delta\}(x, y)\, e^{-i\left(\mathbf{G}_i - \mathbf{G}_j\right)\cdot\mathbf{r}} d^2\mathbf{r}_{\perp}. \tag{9}$$

Substituting Supplementary Eqs. (7), (8) into Supplementary Eq. (6), we obtain linear equations for the Fourier components of the electromagnetic field

$$k_0^2\varepsilon\psi^{\uparrow}_{\mathbf{G}} - \sum_{G'} m_{\mathbf{G}-\mathbf{G}'}(\mathbf{q}+\mathbf{G})\cdot(\mathbf{q}+\mathbf{G}')\psi^{\uparrow}_{\mathbf{G}'} = i\sum_{G'} \Delta_{\mathbf{G}-\mathbf{G}'}\left[(q_x+G_x)(q_y+G'_y) - (q_x+G'_x)(q_y+G_y)\right]\psi^{\uparrow}_{\mathbf{G}'}. \tag{10}$$

We consider the dispersion of the modes near the K (K$'$) points. These are at the corners of the crystal Brillouin zone and correspond to the Bloch wave vectors $\mathbf{K}_{\pm} = K(\pm 1, 0, 0)$, where $K = \dfrac{4\pi}{3a}$, and a is the lattice constant.

For the K (K$'$) valleys $\mathbf{q}+\mathbf{G}=\mathbf{K}_\pm+\delta\mathbf{k}+\mathbf{G}\equiv\mathbf{k}+\delta\mathbf{k}$, where $\delta\mathbf{k}$ is a small detuning, and $\mathbf{G}$ is the reciprocal vector. We truncate the basis to the first three plane waves with the wavevectors $\mathbf{k}_{1,2,3}=\mathbf{K}_\pm+\mathbf{G}_{0,1,2}$ each rotated by $2\pi/3$ with respect to one another and corresponding to the vectors $\mathbf{G}_0=(0,0)$, $\mathbf{G}_1=K\left(\mp\frac{3}{2},-\frac{\sqrt{3}}{2}\right)$, $\mathbf{G}_2=K\left(\mp\frac{3}{2},\frac{\sqrt{3}}{2}\right)$. Thus, to describe the formation of the bands, we leave only the leading contributions from three Γ points nearest to K (K$'$) with $\mathbf{k}_1=(\pm K,0)$, $\mathbf{k}_2=K\left(\mp\frac{1}{2},-\frac{\sqrt{3}}{2}\right)$, $\mathbf{k}_3=K\left(\mp\frac{1}{2},\frac{\sqrt{3}}{2}\right)$. Next, we apply the $k\cdot p$ approximation, keeping only the first order in $\delta\mathbf{k}$, $(\mathbf{k}_i+\delta\mathbf{k})\cdot(\mathbf{k}_j+\delta\mathbf{k})\approx\mathbf{k}_i\cdot\mathbf{k}_j+\delta\mathbf{k}\cdot(\mathbf{k}_i+\mathbf{k}_j)$. Neglecting terms of higher order, we obtain a set of three equations, which can be written in matrix form as

$$k_0^2\varepsilon\vec{\psi}^\uparrow=\left(\hat{m}+\hat{\theta}_\mathrm{b}\right)\vec{\psi}^\uparrow, \tag{11}$$

where the column vector is $\vec{\psi}^\uparrow=(\psi^\uparrow_{\mathbf{G}_0},\psi^\uparrow_{\mathbf{G}_1},\psi^\uparrow_{\mathbf{G}_2})^\mathrm{T}$, and the 3×3 matrices are given by

$$\hat{m}=K^2\begin{pmatrix} m_0 & -\frac{1}{2}m_1 & -\frac{1}{2}m_1\\ -\frac{1}{2}m_1 & m_0 & -\frac{1}{2}m_1\\ -\frac{1}{2}m_1 & -\frac{1}{2}m_1 & m_0\end{pmatrix}+K\delta k_x\begin{pmatrix} 2m_0 & \frac{1}{2}m_1 & \frac{1}{2}m_1\\ \frac{1}{2}m_1 & -m_0 & -m_1\\ \frac{1}{2}m_1 & -m_1 & -m_0\end{pmatrix}+K\delta k_y\begin{pmatrix} 0 & -\frac{\sqrt{3}}{2}m_1 & \frac{\sqrt{3}}{2}m_1\\ -\frac{\sqrt{3}}{2}m_1 & -\sqrt{3}m_0 & 0\\ \frac{\sqrt{3}}{2}m_1 & 0 & \sqrt{3}m_0\end{pmatrix}, \tag{12}$$

$$\hat{\theta}_\mathrm{b}=i\Delta_1K^2\frac{\sqrt{3}}{2}\begin{pmatrix}0 & -1 & 1\\ 1 & 0 & -1\\ -1 & 1 & 0\end{pmatrix}. \tag{13}$$

The coefficients m_0, m_1 and Δ_1 are the Fourier components calculated from Eq. (9). To diagonalize the Hamiltonian at high symmetry points K(K$'$) in the Brillouin zone, we perform the unitary transformation $\hat{H}=U\hat{H}_1U^{-1}$ with the matrix

$$U=\frac{1}{\sqrt{3}}\begin{pmatrix}1 & 1 & 1\\ 1 & e^{-2i\pi/3} & e^{2i\pi/3}\\ 1 & e^{2i\pi/3} & e^{-2i\pi/3}\end{pmatrix}. \tag{14}$$

After excluding the row describing a singlet state, we obtain the effective 2×2 Hamiltonian in the subspace of the doublet states. The same procedure can be repeated for the opposite pseudo-spin. Finally, we arrive at the Hamiltonian for two spin configurations

$$H^{\uparrow,\downarrow}=\begin{pmatrix}\Omega_0\hat{\sigma}_0+V\left(\delta k_x\hat{\sigma}_x+\delta k_y\hat{\sigma}_y\right)\pm m_\mathrm{B}\hat{\sigma}_z & 0\\ 0 & \Omega_0\hat{\sigma}_0+V\left(-\delta k_x\hat{\sigma}_x+\delta k_y\hat{\sigma}_y\right)\mp m_\mathrm{B}\hat{\sigma}_z,\end{pmatrix}, \tag{15}$$

where the unperturbed frequency $\Omega_0=K^2\left(m_0+\frac{1}{2}m_1\right)$, velocity $V=K(m_0-m_1)$, and mass terms due to bianisotropy $m_\mathrm{B}=\frac{3}{2}K^2\Delta_1$. The Hamiltonian in Supplementary Eq. (15) can be written in a compact form

$$H=\Omega_0+V\delta k_x\hat{\tau}_z\hat{\sigma}_x+V\delta k_y\hat{\sigma}_y+m_\mathrm{B}\hat{S}_z\hat{\tau}_z\hat{\sigma}_z, \tag{16}$$

where the Pauli matrices $\hat{S},\hat{\tau}$ and $\hat{\sigma}$ represent subspaces of spin, valley and double states, respectively. From Supplementary Eq. (16), we extract the spin Chern number at each valley by calculating the Berry flux and integrating it over the local valley

$$C^\uparrow_{K,K'}=\frac{1}{2},C^\downarrow_{K,K'}=-\frac{1}{2}. \tag{17}$$

Valley index created in triangular lattice

The triangle-lattice described in the main text also has broken inversion symmetry in the direction parallel to the metal waveguide (x direction). We still assume that the magnetic permeability and electric permittivity have the same magnitude, and

we follow the same procedure as before, except there is no bianisotropic term in Maxwell's equations. The linear equations for the Fourier components are written with the index of pseudo-spin is omitted

$$\sum_{G'} m_{\mathbf{G}-\mathbf{G}'}(\mathbf{q}+\mathbf{G})\cdot(\mathbf{q}+\mathbf{G}')\psi_{\mathbf{G}'} = k_0^2\varepsilon\psi_{\mathbf{G}}\,. \tag{18}$$

To account for the triangular lattice, we introduce three small circular perturbations of radius r_2 at the outer edge of the unperturbed rod of radius r_1 with centers along the direction of the vectors $\mathbf{d}_1 = d_0\left(-\frac{\sqrt{3}}{2}, -\frac{1}{2}\right)$, $\mathbf{d}_2 = d_0(0,1)$, $\mathbf{d}_3 = d_0\left(\frac{\sqrt{3}}{2}, -\frac{1}{2}\right)$, which are parallel to the reciprocal vectors $\mathbf{G}_{10}, \mathbf{G}_{21}$ and $\mathbf{G}_{02}$, respectively Here, $d_0 = r_1 + r_2$. Accordingly, we write the spatial distribution as

$$m(\mathbf{r}_\perp) = m_{\text{env}} + (m_{\text{rod}} - m_{\text{env}})\left(\Pi(\mathbf{r}_\perp, r_1) + \sum_{n=1}^{3}\Pi(\mathbf{r}_\perp - \mathbf{d}_n, r_2)\right), \tag{19}$$

where function $\Pi(\mathbf{r}_\perp, r_{1,2})$ are selection functions for circles with the radii $r_{1,2}$, values m_{env} and m_{rod} corresponding to the indices of the environment and the interior of cylinders. The Fourier coefficients m_{ij} can then be written as

$$m_{ij} = \left[\begin{array}{ll} m_0 + 3\Delta m_0(r_2), & i = j \\ m_1 + \Delta m_1(r_2)\sum_{n=1}^{3} e^{-i(\mathbf{G}_i - \mathbf{G}_j)\mathbf{d}_n}, & i \neq j \end{array}\right.. \tag{20}$$

We obtain the 3×3 eigenvalue problem

$$k_0^2\varepsilon\vec{\psi} = (\hat{m} + \hat{m}_{\text{tri}})\,\vec{\psi}, \tag{21}$$

where the column-vector $\vec{\psi} = (\psi_{\mathbf{G}_0}, \psi_{\mathbf{G}_1}, \psi_{\mathbf{G}_2})^{\text{T}}$, and the $\hat{m}$ matrices have the same form as in Supplementary Eqs. (12), and $\hat{m}_{\text{tri}}$ is defined as

$$\hat{m}_{\text{tri}} = iK^2\begin{pmatrix} 3\Delta m_0 & -\frac{1}{2}\Delta m_1\left(e^{i\varphi} + 2e^{-i\varphi/2}\right) & -\frac{1}{2}\Delta m_1\left(e^{-i\varphi} + 2e^{i\varphi/2}\right) \\ -\frac{1}{2}\Delta m_1\left(e^{-i\varphi} + 2e^{i\varphi/2}\right) & 3\Delta m_0 & -\frac{1}{2}\Delta m_1\left(e^{i\varphi} + 2e^{-i\varphi/2}\right) \\ -\frac{1}{2}\Delta m_1\left(e^{i\varphi} + 2e^{-i\varphi/2}\right) & -\frac{1}{2}\Delta m_1\left(e^{-i\varphi} + 2e^{i\varphi/2}\right) & 3\Delta m_0 \end{pmatrix}, \tag{22}$$

where $\varphi = \sqrt{3}Kd_0$. Repeating the same procedure as in subsection A, we arrive at the Hamiltonian for two spin configurations

$$H = \Omega_0 + V\delta k_x\hat{\tau}_z\hat{\sigma}_x + V\delta k_y\hat{\sigma}_y + m_{\text{I}}\hat{\sigma}_z, \tag{23}$$

where mass term due to breaking inversion symmetry is $m_{\text{I}} = K^2\sqrt{3}\Delta m_1\sin\frac{\varphi}{2}\left(\cos\frac{\varphi}{2} - 1\right)$, and other terms are the same expression as shown in subsection A. the Chern numbers at each valley are calculated to be

$$C_{K,K'} = \pm\frac{1}{2}. \tag{24}$$

At the domain wall between the rod-lattice and the triangle-lattice, the bulk-interface correspondence can be applied independently at each valley. In Fig.2a, $\Delta C_K^{\downarrow} = -1, \Delta C_{K'}^{\uparrow} = 1$ indicates that there are two nontrivial counter-propagating edge modes in distinct spin-valley sectors. The sign of ΔC indicates the propagating direction. Similar results are indicated in Fig.2b.

Supplementary Note 2 – Optical-scale design of the QSH system

To realize an optical QSH structure, we designed a system based on germanium ($\varepsilon_r = 16$) which can be produced with use of conventional nanofabrication techniques. The structure is a triangular array of germanium nano-disks with dimensions selected so that the array supports two doubly degenerate Dirac cones stemming from electric and magnetic dipolar modes at both the K and K' points of the Brillouin zone. The band structure plotted in Supplementary Figure 1(a) reveals two pairs of Dirac bands whose degeneracy effectively emulates the spin degree of freedom. In order to introduce coupling between the electric and magnetic modes, which gives the bianisotropic response, we reduce the out-of-plane inversion symmetry by introducing a circular notch on one of the flat faces of the cylinders. This gives rise to a complete photonic band gap (Supplementary Figure 1(b)). The resultant top and bottom bands do not cross anywhere in the Brillouin zone. This implies that the topological properties are completely defined by the hybridization of the magnetic and electric Dirac bands near the K and K' points. In this preliminary optical design, we neglect the effect of the substrate, which can be valid for the case of a structure fabricated on top of a thin membrane, such as a 20-nm-thick SiN membrane.

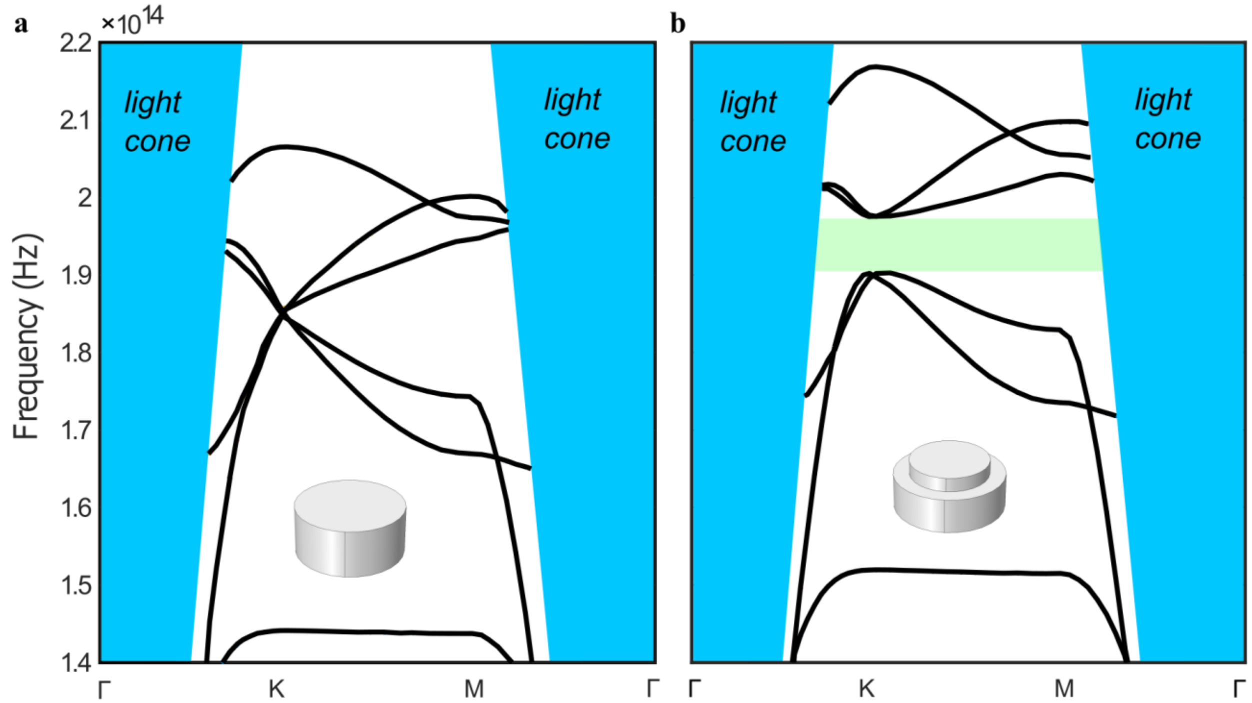

Supplementary Figure 1| Band structure of all-dielectric optical metamaterials. (**a**) Numerically calculated photonic band structure of spin-valley degenerate germanium-based all-dielectric optical metamaterial. (**b**) Photonic band structure of optical metamaterial design with magneto-electric coupling. Insets in panels show the corresponding dielectric nanodisks of both metasurfaces. The light-green shaded area illustrates the spectral bandwidth of the topological band gap. The position of the radiative continuum (above the light cone) is marked by the blue shaded areas. The geometrical parameters are: the period is 700 nm, large disk radius and height are 263 nm and 168 nm, small disk radius and height are 188 nm and 68 nm, respectively.
